# Adalimumab treatment in Juvenile Idiopathic Arthritis (JIA) patients with special focus on Uveitis

**DOI:** 10.1186/1546-0096-9-S1-P317

**Published:** 2011-09-14

**Authors:** R Joos, C Wouters, J Dehoorne, D Elewaut

**Affiliations:** 1 Centre for pediatric rheumatology, Gent, Belgium; 2 Dept of pediatry, Leuven, Belgium; 3 Dept of rheumatology, ZNA, Antwerp, Belgium

## Objectives

We performed an observational study to evaluate the efficacy of adalimumab treatment of uveitis and arthritis in patients with juvenile idiopathic arthritis.

## Results

23 JIA patients with polyarticular course (initial diagnosis: 12 oligo , 2 systemic, 2 psoriatic arthritis, 4 ERA and 3 polyarticular) were treated with adalimumab. Uveitis was found in 14 patients. (SoJIA: 2/2; OligoJIA 12/12). The results of the first six months are communicated.

Ocular: During the first six months of adalimumab treatment no new uveitis episodes occurred. In all patients visual acuity was impaired during the active uveitis phase at the start of adalimumab treatment. Some patients (5/14) recovered sight completely, but in the others impairment of visual acuity remained. The anterior chamber cellularity decreased dramatically in the first 8 weeks. Yet in three patients a low number of white blood cells was still seen after 24 weeks.

Articular: the number of active joints ranged from 0 to 5 (mean 1,30, median 2) at start, at 3 months mean = 0,70 and median = 1, range 0 - 4 and at six months mean = 0,22, median 0, range 0 - 2. The number of joints with LOM ranged from 0 – 5 (mean 1,43, median 1) at start, mean 0,91 after 3 months (median 0, range 0 -5) and mean 0,39 after 6 months (median 0, range 0 – 4).

## Conclusion

Despite the limitations of a relatively small patient group adalimumab treatment shows to be beneficial in JIA patients as well on the ocular disease as on the arthritis. Especially for uveitis we need larger long-term prospective randomized series to draw definite conclusions.

